# Targeting tumors with a killer-reporter adenovirus for curative fluorescence-guided surgery of soft-tissue sarcoma

**DOI:** 10.18632/oncotarget.3811

**Published:** 2015-04-14

**Authors:** Shuya Yano, Shinji Miwa, Hiroyuki Kishimoto, Fuminari Uehara, Hiroshi Tazawa, Makoto Toneri, Yukihiko Hiroshima, Mako Yamamoto, Yasuo Urata, Shunsuke Kagawa, Michael Bouvet, Toshiyoshi Fujiwara, Robert M. Hoffman

**Affiliations:** ^1^ AntiCancer, Inc., San Diego, CA, USA; ^2^ Department of Surgery, University of California San Diego, CA, USA; ^3^ Department of Gastroenterological Surgery, Okayama University, Graduate School of Medicine, Dentistry and Pharmaceutical Sciences, Okayama, Japan; ^4^ Center for Innovative Clinical Medicine, Okayama University Hospital, Okayama, Japan; ^5^ Oncolys BioPharm Inc., Tokyo, Japan

**Keywords:** soft tissue sarcoma, nude mice, fluorescence-guided surgery (FGS), adenovirus, OBP-401

## Abstract

Fluorescence-guided cancer has not yet been shown to be curative due to residual microscopic disease. Human fibrosarcoma HT1080 expressing red fluorescent protein (RFP) was implanted orthotopically in the quadriceps femoris muscle of nude mice. The tumor-bearing mice were injected with high and low-dose telomerase-dependent, green fluorescent protein (GFP)-containing adenovirus OBP-401, which labeled the tumor with GFP. Fluorescence-guided surgery (FGS) or bright light surgery (BLS) was then performed. OBP-401 could label soft-tissue sarcoma (STS) with GFP *in situ*, concordant with RFP. OBP-401-based FGS resulted in superior resection of STS in the orthotopic model of soft-tissue sarcoma, compared to BLS. High-dose administration of OBP-401 enabled FGS without residual sarcoma cells or local or metastatic recurrence, due to its dual effect of cancer-cell labeling with GFP and killing. High-dose OBP-401 based-FGS improved disease free survival (*p* = 0.00049) as well as preserved muscle function compared with BLS. High-dose OBP-401-based FGS could cure STS, a presently incurable disease. Since the parent virus of OBP-401, OBP-301, has been previously proven safe in a Phase I clinical trial, it is expected the OBP-401-FGS technology described in the present report should be translatable to the clinic in the near future.

## INTRODUCTION

Fluorescence-guided surgery (FGS) of cancer is an area of broad and intense interest [1]. In the clinic, sentinel lymph nodes have been labeled by the near-infrared (NIR) fluorescing dye indocyanine [2]. However, indocyanine does not specifically label tumor cells. 5-aminolevulinic acid (5-AA), a precursor of hemoglobin has been used to label malignant glioma with significant progression-free survival benefit, but was not curative [3]. Folate conjugated to fluorescein isothiocyanate (FITC) was used for targeting folate receptor–α (FR-α) in ovarian cancer patients whereby deposits less than 1 mm could be resected but was not shown to be curative [4]. Urano *et al*. used fluorescence-guided laparoscopy to visualize and remove tumors illuminated by the probe, γGlu-HMRG. invasive human ovarian cancer, which in a mouse model, expresses the probe-activating GTT enzyme, but cures were not demonstrated [5].

We previously reported the effectiveness of FGS to improve outcomes in retroperitoneal-implanted nude mouse model of human fibrosarcoma, expressing green fluorescent protein (GFP). Although tumor recurrence was reduced and disease-free survival (DFS) increased, the procedure was not curative [6].

In another study from our laboratory, after FGS of a human pancreatic cancer cell line expressing green fluorescent protein (GFP) in an orthotopic nude-mouse model, the surgical resection bed was irradiated with UVC. FGS-UVC-treated mice had increased DFS and overall survival (OS) compared to FGS-only treated mice; with DFS lasting at least 150 days, indicating the animals were cured.

GFP has been used for labeling tumors *in situ* for FGS. Kishimoto *et al*. [7] selectively-labeled tumors with GFP using a telomerase-dependent adenovirus (OBP-401) that expresses the *gfp* gene only in cancer cells, which generally express the telomerase enzyme in contrast to normal cells. The labeled tumors could then be resected under fluorescence guidance. Tumors that recurred after FGS maintained GFP expression [8]. Because the recurrent cancer cells stably express GFP, detection of cancer recurrence and metastasis is also possible with OBP-401 GFP labeling, in contrast to fluorescent-antibody or other non-genetic labeling. Because tumors of all types express telomerase, the genetic labeling method that uses a telomerase-dependent adenovirus to deliver GFP specifically to tumors offers the potential of widespread application, since cancers of all types express telomerase.

Approximately 11,000 soft tissue sarcomas (STS) are diagnosed every year in the United States [9-12]. The standard treatment of soft tissue sarcomas comprises wide resection with or without adjuvant radiotherapy and/or chemotherapy. At 5 years, the cumulative probability of local recurrence reported in large series ranges from 12% to 28% [13-17], and the cumulative probability of metastasis ranges from 21% to 40% [13-18]. Wide excision can include muscle, tendon, ligament, bone, and joints which often decease limb function. The presence or absence of residual cancer cells in the surgical area determines local recurrence and prognosis of survival. Local recurrence after adjuvant therapy is mostly resistant to treatment, resulting in poor prognosis [13-17].

We report here FGS in combination with OBP-401 is curative, of an STS in an orthotopic model, with preservation of walking function.

## RESULTS AND DISCUSSION

### GFP-expressing adenovirus OBP-401 labels and kills sarcoma cells *in vitro*

**Figure 1:**
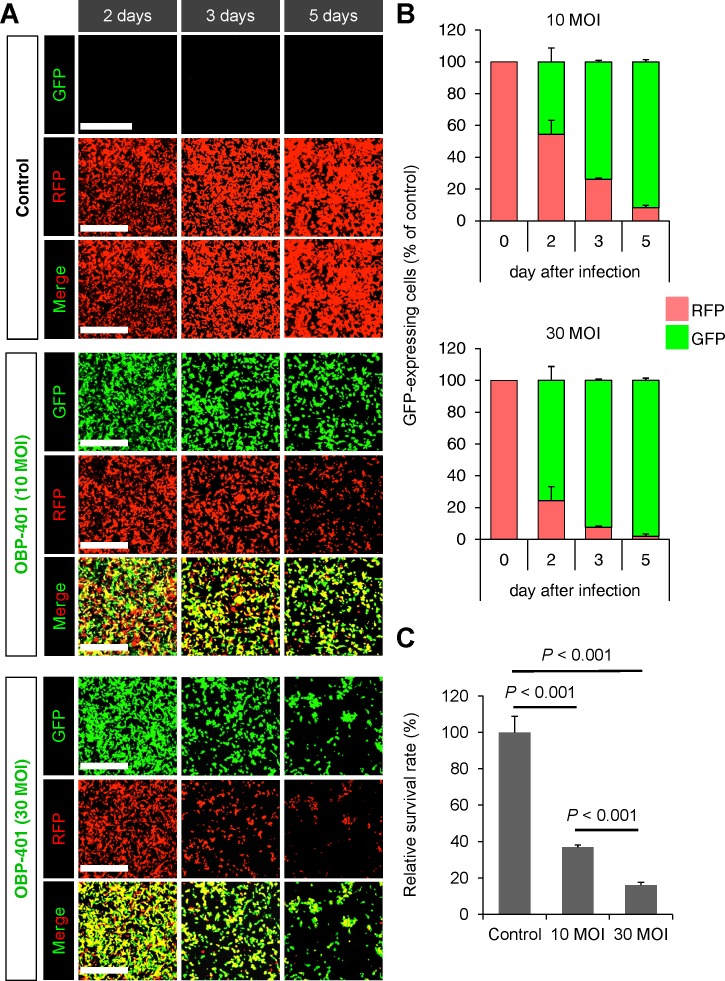
OBP-401 labels soft tissue sarcoma cell line HT1080-RFP with GFP and then kills them *in vitro* HT1080 human fibrosarcoma cells expressing RFP cells are seeded in 6 well plates (1×10^5^ cells well). OBP-401 was added at the indicated multiplicity of infection (MOI) 24 hours after cell seeding. Images were acquired with a FV1000 confocal laser scanning microscope (Olympus). **A**. Representative images of mock-infected HT1080 sarcoma cells (upper). Representative images of HT1080 sarcoma cells 2, 3, and 4 days after infection with OBP-401 at an MOI of 10 (middle). Representative images of HT1080 sarcoma cells 2, 3, and 4 days after infection with OBP-401 at an MOI of 30 (lower). Bar=500 μm. **B**. Histogram shows the frequency of GFP-expressing sarcoma cells at indicated days after infection of OBP-401. **C**. Histogram shows the surviving fraction of HT1080 sarcoma cells 6 days after infection of OBP-401 *in vitro*. Data are shown as average ± SD. N = 5.

Time-lapse imaging demonstrated that OBP-401 labeled RFP-expressing HT1080 sarcoma cells with GFP (Figure 1A). OBP-401 labeled HT1080 cells with GFP in a dose-dependent manner (Figure 1B). GFP fluorescence, after OBP-401 infection of HT1080 cells, became stronger each day from day 2 to day 7 (Figure 1A). OBP-401 also killed HT1080 cells with GFP in a dose-dependent manner (Figure 1C). These data indicated that OBP-401 labeled sarcoma cells with GFP, and could subsequently kill them *in vitro*.

### Orthotopic sarcoma model

An orthotopic sarcoma model was developed with HT1080 RFP cells implanted in the quadriceps femoris muscles. The orthotopically-growing sarcoma cells invaded the quadriceps femoris muscles and femoral bone similar to the clinical course of STS. Tumor growth was visualized by RFP fluorescence.

### Bright-light surgery results in remaining sarcoma cells in the orthotopic model

**Figure 2:**
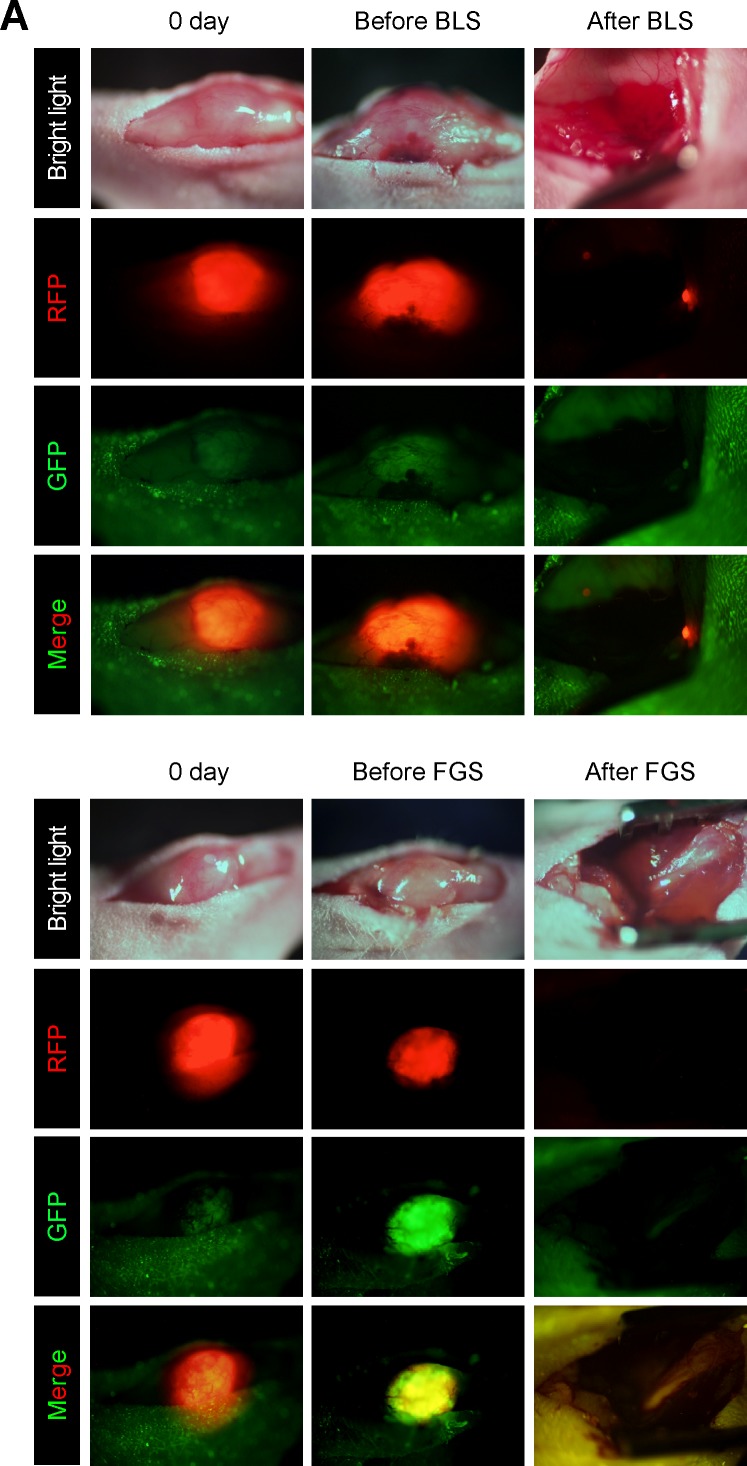

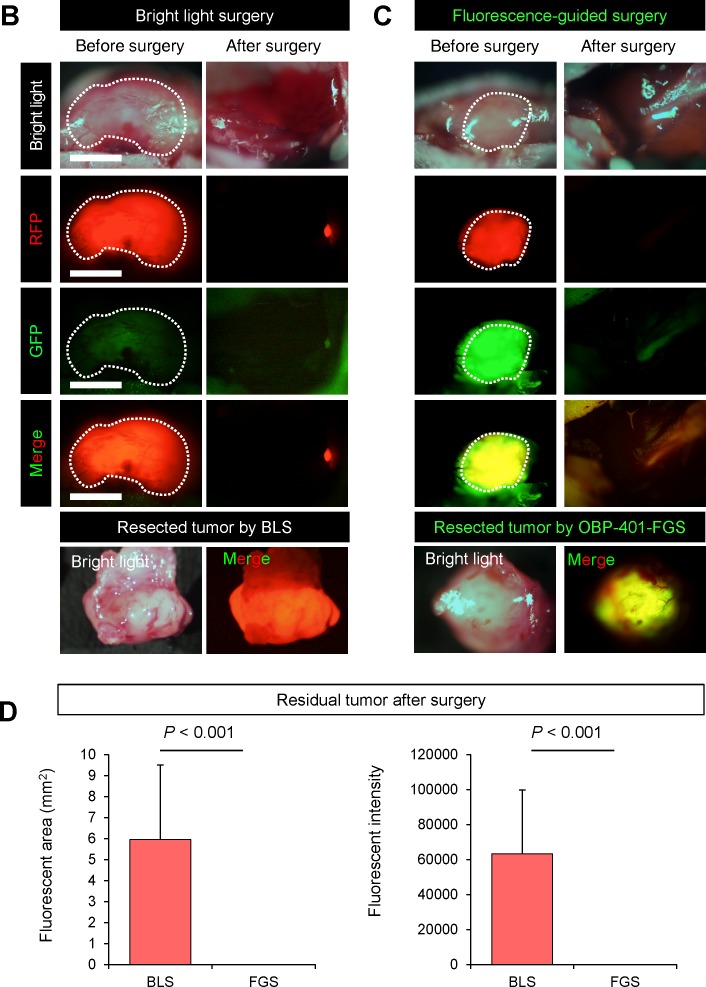
Comparison of OBP-401 based fluorescence-guided surgery with bright-light surgery for orthotopic soft tissue sarcoma For the orthotopic sarcoma model, HT1080 cells (3×10^6^) in Matrigel (BD) were inoculated in the left quadriceps femoris muscle of nude mice (5 weeks old). For fluorescence-guided surgery (FGS), OBP-401 was injected intaratumorally at 1×10^8^ PFU when tumors reached approximately 100 mm^3^ (6 mm diameter). **A**. Representative whole-body images of mock-infected orthotopic sarcoma before and after bright-light surgery (BLS) (upper). Representative whole-body images of orthotopic sarcoma before injection of OBP-401 and before and after OBP-401-based FGS (OBP-401-FGS) (lower). **B**. Representative high-magnification images of RFP-expressing orthotopic sarcoma tumor before and after BLS Bar=7.5 μm. **C**. Representative high-magnification images of OBP-401-GFP labeled, RFP-expressing orthotopic sarcoma before and after OBP-401-FGS. **D**. Bargraphs shows the comparison of fluorescent area between BLS-treated tumors and OBP-401-FGS-treated tumors (*p* = 0.001) (left). Fluorescent area is calculated with ImageJ software. Bargraphs shows the comparison of fluorescence intensity of BLS-treated tumors and OBP-401-FGS-treated tumors (*p* = 0.001) (right). Fluorescence intensity is calculated with ImageJ software. Data are shown as average ± SD. N = 10.

We performed bright-light surgery (BLS) on the orthotopic sarcoma model (Figure 2A). Because the sarcoma invaded muscles and the femoral bone, the tumor margin was invisible under bright light. Extensive RFP-expressing sarcoma cells remained after BLS (Figure 2A and 2D).

### OBP-401-based fluorescence-guided surgery (OBP-401-FGS) of orthotopic sarcoma

The orthotopic tumor growing in the quadriceps femur (100 mm^3^, diameter; 6 mm) was resected 3 days after i.t. injection of OBP-401 at 1×10^8^ PFU (Figure 2A). OBP-401 conferred GFP fluorescence of the orthotopic sarcoma which was sufficiently bright to perform complete resection of the sarcoma (Figure 2C and 2D). Imaging showed that OBP-401 GFP labeling matched the tumor RFP fluorescence (Figure 2C). OBP-401-GFP-based FGS resulted in no detectable residual sarcoma cells (Figure 2C and 2D).

### OBP-401-FGS detects and resects residual sarcoma cells in the orthotopic STS model after BLS

**Figure 3:**
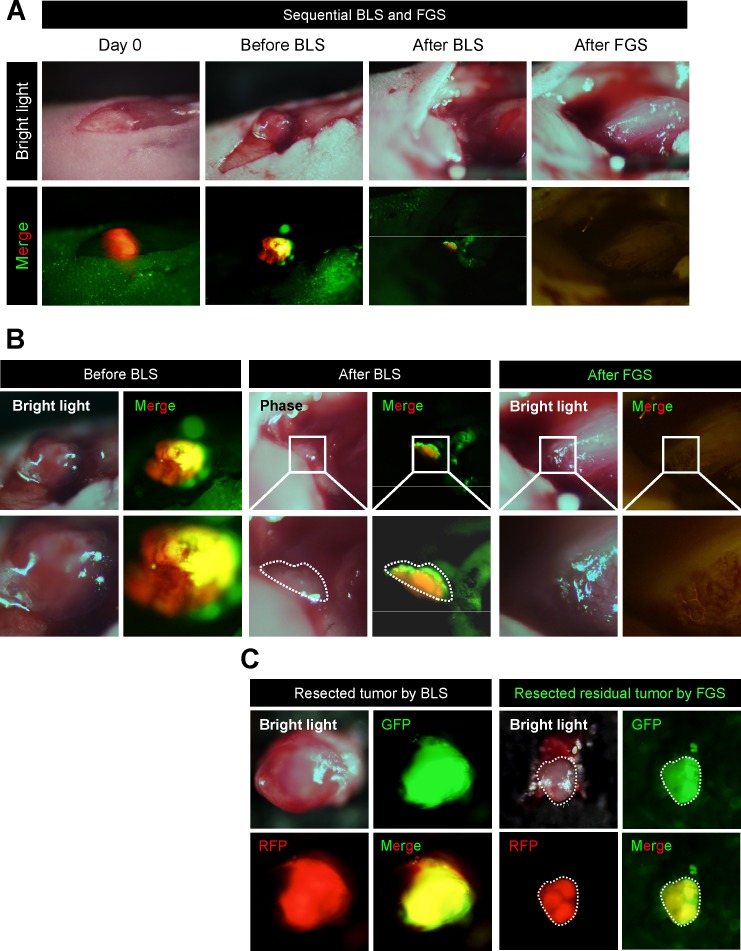
OBP-401-FGS removes residual sarcoma cells after BLS HT1080-RFP cells (3×10^6^) in Matrigel were inoculated in the left quadriceps femoris muscle of nude mice (5 weeks). OBP-401 was injected intaratumorally at 1×10^8^ PFU when tumors reached approximately 100 mm^3^ (6 mm diameter). The OBP-401-labeled orthotopic tumor was resected under bright light, and then residual tumor was resected under fluorescence. **A**. Representative whole-leg images of orthotopic sarcoma before and after BLS and FGS. **B**. Representative whole-tumor images of orthotopic sarcoma before and after BLS and FGS. **C**. Representative images of resected tumor after BLS (left) are after OBP-401-FGS (right).

OBP-401 was intratumorally injected 3 days before BLS in HT1080-RFP tumors growing in the quadriceps femoris muscle (Figure 3A). After BLS, both RFP and GFP fluorescence were detected in the surgical bed (Figure 3B). OBP-401 enabled detection of the residual cancer cells at the single cell level (Figure 3B). After OBP-401-FGS, there were no residual cancer cells (Figure 3B). OBP-401 delineated the precise margin between cancer and normal tissue and whether there were residual cancer cells at the single-cell level (Figure 3C).

### OBP-401 based FGS resulted in recurrence-free surgery

We compared the rate of local recurrence after OBP-401-FGS with BLS alone. Fluorescence imaging showed that 8 of 10 mice that underwent BLS had RFP-expressing, small, hard nodules 3 days after surgery, indicating that these mice had local recurrences (Figure 5A and 5B). In contrast there was no local recurrence in eight mice which received OBP-401-FGS (Figure 5A-5C, Table 1A). These data demonstrated that tumor visualization at the single-cell level by OBP-401 enabled complete resection and prevented local recurrence.

**Table 1A-B:** Local and metastatic recurrence after BLS and low- and high-dose OBP-401-FGS

**A**	**Local recurrence**	**Positive**	**Negative**		
	BLS	8	2	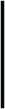 *	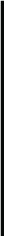 **
	Low-dose OBP-401-FGS	0	9		
	High-dose OBP-401-FGS	0	10		
**B**	**Lung metastasis**	**Positive**	**Negative**		
	BLS	8	2	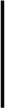 *	
	Low-dose OBP-401-FGS	3	6		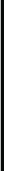 **
	High-dose 010-401-FGS	0	10		

* *p* = 0.040

** *p* = 0.043

### OBP-401-FGS enables minimally invasive, function-preserving surgery for sarcoma

**Figure 4:**
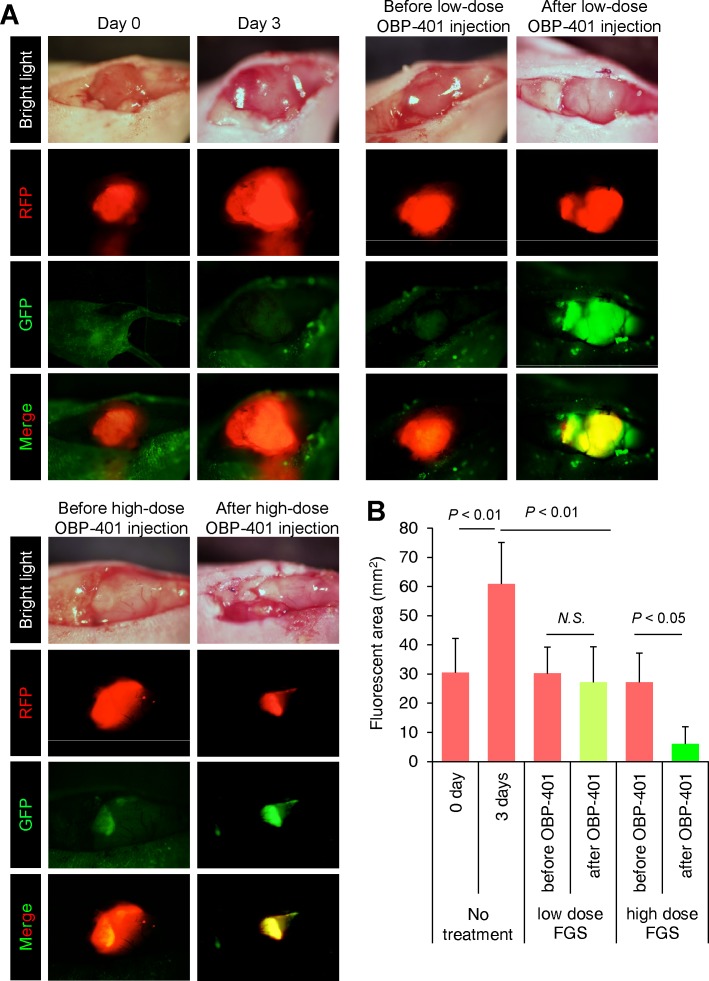

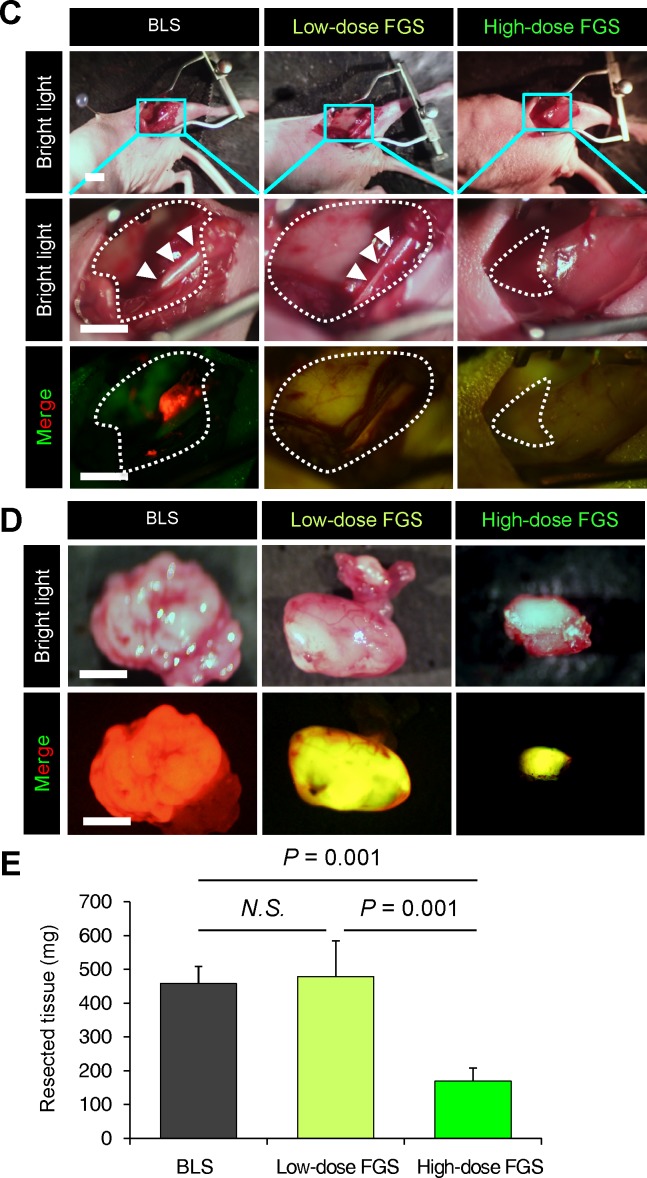

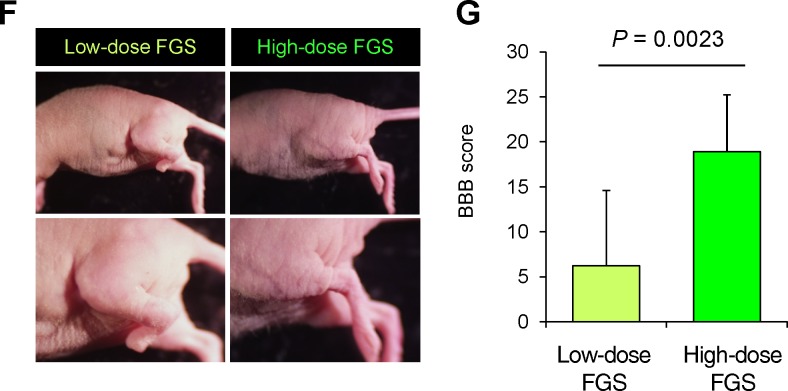
High-dose OBP-401 enables function-preserving surgery for orthotopic soft tissue sarcoma HT1080 cells (3×10^6^) in Matrigel (BD) were inoculated in the left quadriceps femoris muscle of nude mice (5 weeks). OBP-401 was injected intratumorally when tumors reached approximately 100 mm^3^ (6 mm diameter). **A**. Representative whole tumor images of orthotopic sarcoma before and after administration of low-dose OBP-401 (1×10^8^ PFU) and high-dose OBP-401 (at 3×10^8^ PFU). **B**. Bargraph shows the fluorescent area of control tumor and low-dose or high-dose OBP-401-treated tumors. Fluorescent area is calculated with ImageJ software. Data are shown as average ± SD. N = 10. **C**. Representative images of surgical area after BLS and low-dose or high-dose OBP-401-FGS. Dotted areas show surgical area. Arrowheads show tibia. RFP-expression means residual tumor. **D**. Representative images of resected tumor after BLS and low-dose or high-dose OBP-401-FGS. **E**. Bargraph shows the volume of resected tumor with BLS and low-dose or high-dose OBP-401-FGS (lower). Data are shown as average ± SD. N = 10, *p* < 0.05. **F**. Representative image of left leg inoculated with low-dose or high-dose OBP-401-FGS. **G**. Bargraph shows the Basso, Beattie and Bresnahan (BBB) score of leg-function after low-dose OBP-401-FGS and high-dose OBP-401-FGS (right) (*p* = 0.0023).

**Figure 5:**
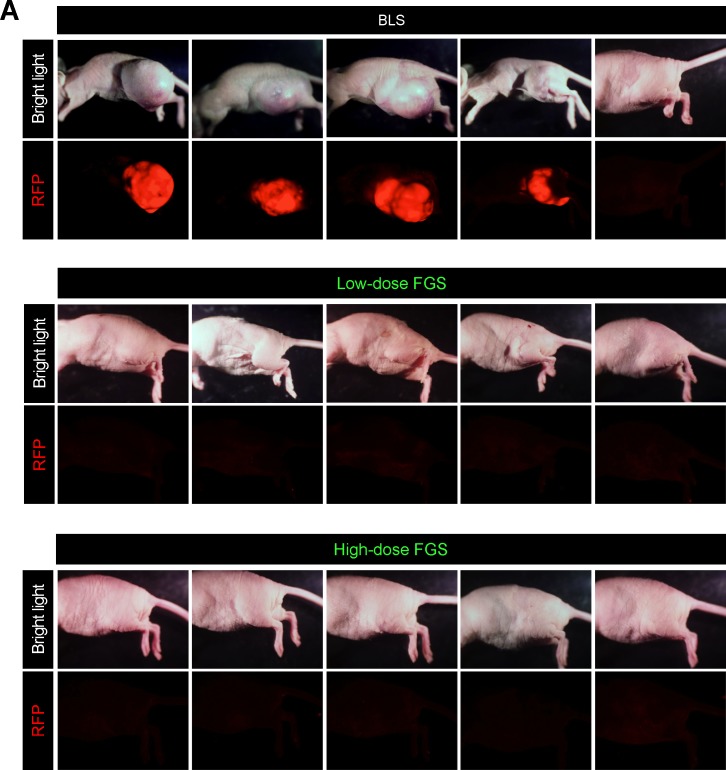

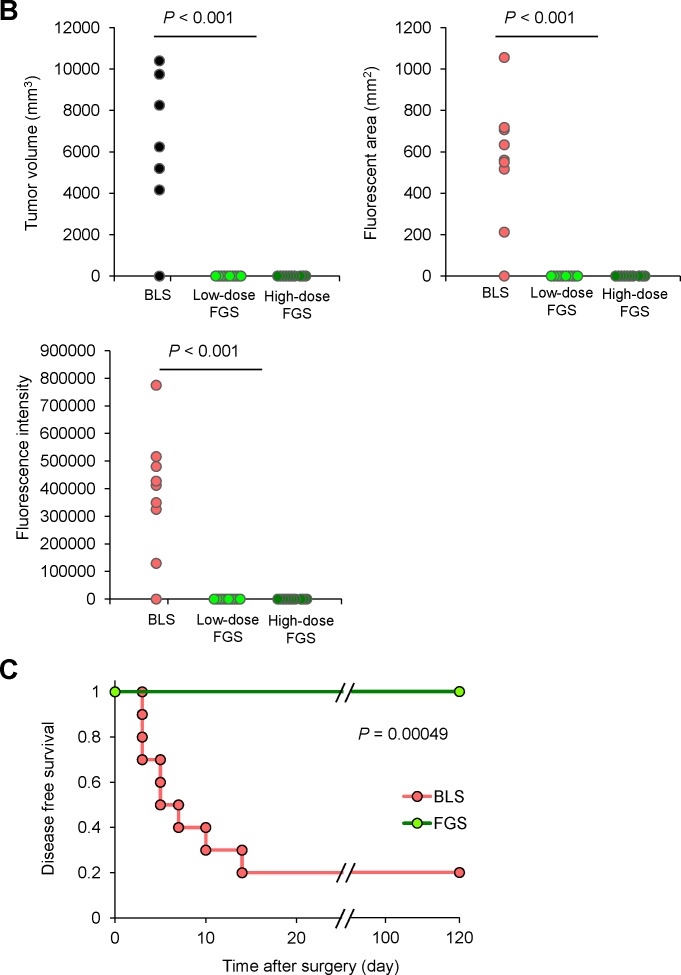
OBP-401-based FGS prevents local recurrence after surgery **A**. Representative whole-body images of orthotopic sarcoma 30 days after BLS (upper), 70 days after low-dose OBP-401-FGS (middle), and 70 days after high-dose OBP-401-FGS (lower). **B**. Comparison of volume of recurrent tumors after BLS, low-dose OBP-401-FGS, or high-dose OBP-401-FGS. Fluorescent area and intensity are calculated with ImageJ software. Data are shown as average ± SD. N = 10. **C**. Kaplan-Meyer curves show the disease-free survival after BLS or low-dose or high-dose OBP-401-FGS (*p* < 0.001).

Next, we confirmed whether high-dose OBP-401 (2×10^8^ PFU) FGS enabled minimally invasive surgery of sarcoma for preservation of muscle compared with BLS or low-dose FGS (Figure 5A). We injected high-dose OBP-401 into tumors in the orthotopic sarcoma model. High-dose injection of OBP-401 significantly reduced the size of tumors and inhibited tibial invasion compared with control tumor or low-dose injection of OBP-401 (Figure 4A and 4B). We minimally resected tumors without residual disease under FGS, after high-dose injection of OBP-401 (Figure 4C). Six of 8 mice lost muscle function after FGS with low-dose OBP-401. In contrast, all mice preserved muscle function after FGS with high-dose OBP-401 (Figure 4D). These data suggested that OBP-401-based FGS enabled muscle function preservation for sarcoma.

### Inhibition of lung metastasis after high-dose OBP-401 and FGS

**Figure 6:**
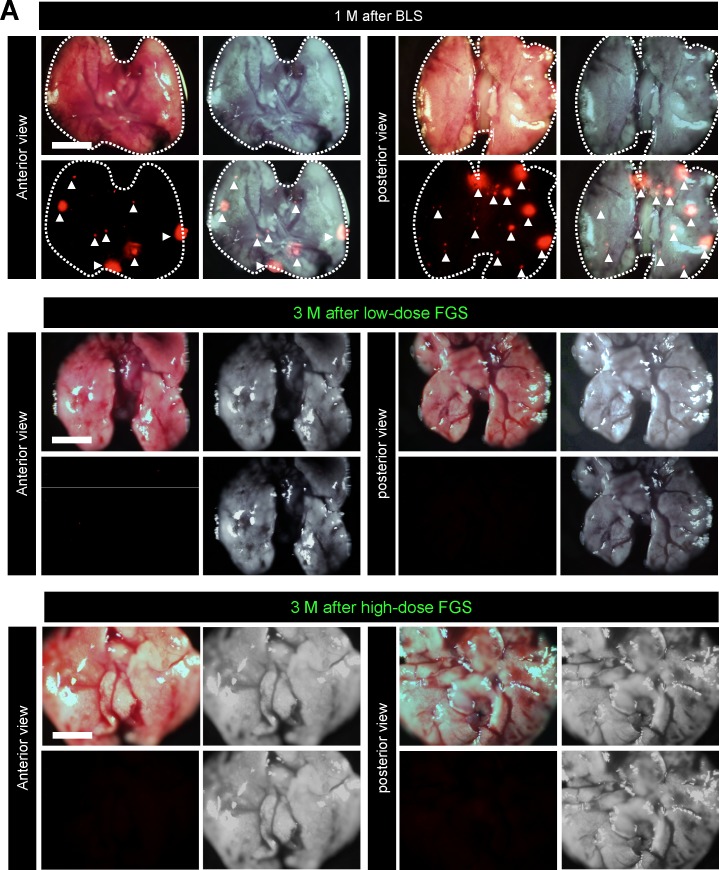

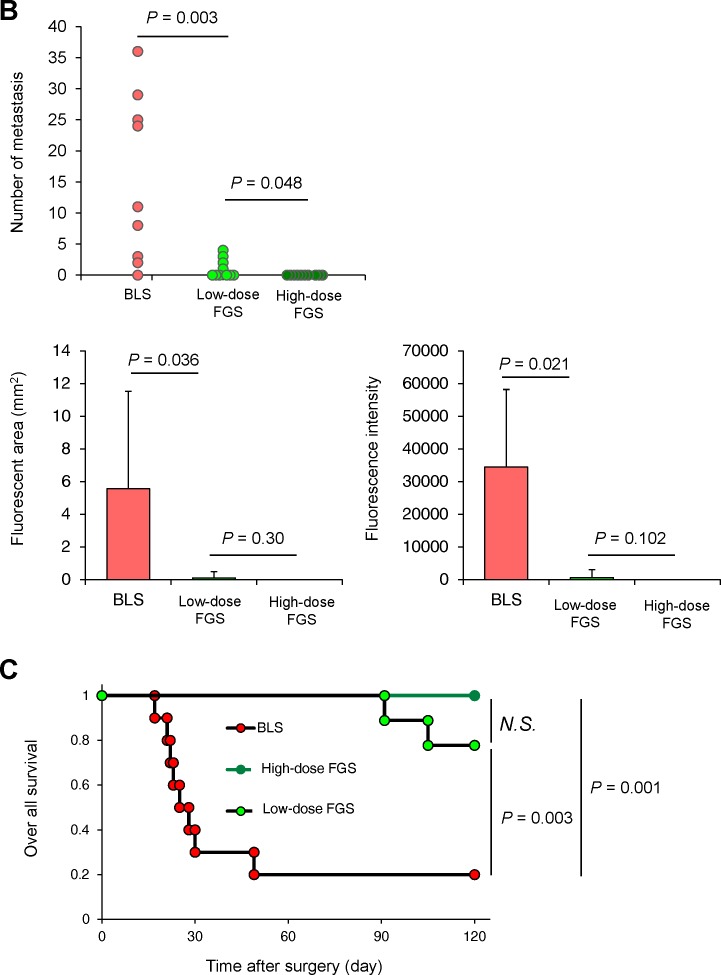
OBP-401-based FGS reduces distant metastasis after surgery To evaluate distant metastasis after BLS or OBP-401-FGS in the orthotopic sarcoma model, mice were sacrificed at the time indicated after surgery and the lungs were imaged. **A**. Representative images of whole lungs in posterior view and anterior view after BLS, after low-dose OBP-401-FGS (middle). Representative images of whole lung in posterior and anterior views after high-dose OBP-401-FGS Red fluorescence (arrow heads)indicates metastasis. Bar=5 mm. **B**. Comparison of number of metastasis in the lung, fluorescencet area and fluorescenceintensity after BLS, low-dose OBP-401-FGS, or high-dose OBP-401-FGS. Fluorescence intensity and fluorescence area are calculated with ImageJ software. **C**. Kaplan-Meyer curves show the overall survival after BLS, low-dose OBP-401-FGS or high-dose OBP-401-FGS.

Eight of 10 mice received BLS had large lung metastases observed at necropsy (Figure 6A-6C). Three of 9 mice which received low-dose OBP-401-FGS had small metastasis in the lung (Figure 6, Table 1B). In contrast, none of the 10 mice which received high-dose OBP-401-FGS had any lung metastasis (Figure 6, Table 1B). High-dose OBP-401-FGS significantly prolonged the overall survival rate compared with BLS alone (Figure 6D). These data indicated that high-dose OBP-401-based FGS controlled local recurrence and distant metastasis, thereby prolonging the survival rate.

Labeling tumors with OBP-401 has none of the weaknesses of non-genetic labeling, particularly loss of label over time and limited expression of the marker used for labeling. OBP-401 may be a general method for labeling tumors that express telomerase, which are the vast majority, and could have broad application for FGS.

The results of the present study suggest that OBP-401-based FGS has clinical potential. An appropriate starting point would be sarcoma which is often superficial and amenable to i.t. injection of OBP-401 as was done in the present study, thereby, reducing potential for systemic toxicity. Administration via the i.t. route enables the potential of simple fluorescence imaging of the tumor with portable equipment [19] even in the patient to determine optimal labeling time, as well as dose, before FGS.

The present study used both low-dose (1×10^8^ PFU) and high-dose (3×10^8^ PFU) OBP-401. Clinical studies can start with low-dose OBP-401 and proceed to high-dose after safety is demonstrated.

A Phase I clinical trial of i.t. injection of OBP-301, the parent of OBP-401, in patients with advanced solid tumors was well tolerated [20]. Subsequent clinical studies could evaluate systemic administration of OBP-401 to target existing metastasis.

The tumor-targeting technology described in the present report can be used along with previously-developed tumor-targeting strategies [21-28].

## MATERIALS AND METHODS

### GFP-expressing telomerase-specific adenovirus

In OBP-401 the promoter element of the human telomerase reverse transcriptase (*hTERT*) gene drives the expression of E1A and E1B genes, linked to an internal ribosome entry site, for selective replication only in cancer cells. The *GFP* gene is driven by the CMV promoter inserted in OBP-401 [29].

### Cell line and cell culture

The human sarcoma cell line HT1080 expressing RFP (HT1080-RFP) [30] was maintained and cultured in DMEM medium with 10% fetal bovine serum (FBS) and 5% penicillin/streptomycin.

### *In vitro* or *ex vivo* imaging

Time-course images of OBP-401 labeled HT1080 sarcoma were acquired with an FV1000 confocal laser-scanning microscope (Olympus, Tokyo, Japan) [31]. For whole-body or whole-tumor imaging, the OV100 Small Animal Imaging System (Olympus), was used [32].

### Animal experiments

Athymic (*nu/nu)* nude mice (AntiCancer, Inc., San Diego) were kept in a barrier facility under HEPA filtration. Mice were fed with an autoclaved laboratory rodent diet (Tecklad LM-485, Western Research Products). All animal studies were conducted in accordance with the principles and procedures outlined in the National Institutes of Health Guide for the Care and Use of Laboratory Animals under Assurance Number A3873–01.

### Orthotopic sarcoma model

RFP-expressing HT1080 cells (3 × 10^6^) suspended in Matrigel (20 uL) were inoculated into the left quadriceps femoris muscle of 5 weeks old female athymic nude mice [33]. Tumor progression was monitored by noninvasive fluorescence imaging (OV100).

### OBP-401 based fluorescence-guided surgery (OBP-401-FGS)

All animal procedures were performed under anesthesia using s.c. administration of a ketamine mixture [10 μl ketamine HCl, 7.6 μ;l xylazine, 2.4 μ;l acepromazine maleate, and 10 μl PBS]. Orthotopic STS tumors labeled with GFP by OBP-401 were observed with noninvasive fluorescence imaging (OV100) before FGS was performed. After surgery, the presence of cancer cells was observed with the OV100. If there were residual cancer cells, an additional resection was performed.

### Statistical analysis

Data are shown as means ± SD. For comparison between two groups, significant differences were determined using the Student's t-test. For comparison of more than two groups, statistical significance was determined with a one-way analysis of variance (ANOVA) followed by a Bonferroni multiple group comparison test. Pearson chi-square analysis was used to compare the rate of local recurrence between BLS and OBP-401-FGS. Statistical analysis for disease-free survival and over-all survival was performed using the Kaplan-Meier test along with log-rank test. Pearson chi-square analysis was used to evaluate the rate of local recurrence and lung metastasis between BLS and OBP-401-FGS and the extent of limb preservation between low-dose OBP-401-FGS and high-dose OBP-401-FGS. *P* values of < 0.05 were considered significant.
